# Iatrogenic Intrathoracic Insufflation During Robot-Assisted Laparoscopic Paraoesophageal Hernia Repair with Toupet Fundoplication

**DOI:** 10.7759/cureus.83604

**Published:** 2025-05-06

**Authors:** Rahul Venna, Tomohiro Yamamoto, Satoshi Yamamoto

**Affiliations:** 1 Anesthesiology, University of Texas (UT) Medical Branch Galveston, Galveston, USA; 2 Medicine, Gunma University School of Medicine, Maebashi, JPN

**Keywords:** anesthesia, chest tube, insufflation, pneumothorax, tension pneumothorax

## Abstract

Minimally invasive surgery, including robotic-assisted laparoscopic procedures, has largely replaced open surgery due to its numerous benefits. However, this approach introduces unique complications such as intraoperative pneumothorax, particularly during upper gastrointestinal (GI) surgeries near the diaphragmatic hiatus. We report the case of a 50-year-old male patient undergoing robotic paraesophageal hernia repair with Toupet fundoplication who developed a tension pneumothorax intraoperatively. Clinical signs included rising peak airway pressures, progressive hypoxemia, and absent right-sided breath sounds. Rapid recognition and immediate chest tube decompression restored ventilation and prevented hemodynamic collapse. The patient recovered without major complications and was discharged on postoperative day two. Overall, intrathoracic insufflation with tension physiology during laparoscopic upper GI surgery is a rare and potentially life-threatening complication. This case highlights the importance of anesthetic vigilance, prompt recognition of ventilatory changes, and swift multidisciplinary intervention to ensure optimal outcomes.

## Introduction

Laparoscopic fundoplication is a widely adopted surgical procedure for the treatment of gastroesophageal reflux disease (GERD) [1]. The two most common forms of this surgery are Nissen fundoplication, a 360-degree wrap of the gastric fundus around the lower esophagus, and Toupet fundoplication, which is a similar 270-degree wrap [1]. This minimally invasive technique reinforces the lower esophageal sphincter [2]. The procedure is acclaimed for its effectiveness in alleviating reflux symptoms and promoting esophageal healing [3]. However, complications may arise during antireflux surgery, one notable example being intrathoracic insufflation [2, 4].

Intrathoracic insufflation refers to the inadvertent introduction of insufflation gas into the thoracic cavity during laparoscopic procedures. This phenomenon can occur due to inadvertent perforation or misplaced instrumentation. Intraoperative complications during laparoscopic Nissen fundoplication, particularly those involving airway or thoracic injury, highlight the procedural vulnerability of this anatomically complex region where even minor disruptions can lead to significant cardiopulmonary compromise, procedural delays, and increased risk of both acute and prolonged postoperative complications [5]. 

In laparoscopic Nissen fundoplication, carbon dioxide (CO₂) is typically used for insufflation to create pneumoperitoneum, which expands the abdominal cavity and provides better visualization and access to the surgical site. Despite the benefits, accidental entry of CO₂ into the thoracic cavity can result in pneumothorax, subcutaneous emphysema, or mediastinal emphysema [6]. These conditions may lead to hemodynamic instability and respiratory distress, necessitating immediate intervention to prevent adverse outcomes. 

We introduce a complex case of abrupt increase in peak pressures, end-tidal CO_2_ (EtCO_2_), and desaturation that proved difficult to rectify; following an accurate diagnosis and the insertion of a chest tube, the patient's condition was successfully restored. 

## Case presentation

A 50-year-old male patient with obesity and a history of GERD, well-controlled on proton pump inhibitors (PPIs), presented for an elective robotic-assisted laparoscopic hiatal hernia repair with Toupet fundoplication. Preoperative evaluation revealed no significant cardiopulmonary or neurologic comorbidities. Blood pressure readings over the prior year varied, with occasional elevations, but the patient had no known cardiovascular events and remained asymptomatic. 

 Pre-anesthesia evaluation categorized the patient as American Society of Anesthesiologists (ASA) Class III due to uncontrolled hypertension with systolic blood pressures exceeding 150 mmHg and obesity with a BMI of 32.86 kg/m². Physical examination was notable for chipped dentition and a Mallampati score of III. General anesthesia was induced with propofol 480 mg, and the airway was secured via oral endotracheal intubation using a Macintosh size 4 blade (IntuBrite, Salter Labs, Arvin, CA, USA) and a 7.5 mm cuffed tube. Intubation was achieved on the first attempt with confirmation by capnometry and auscultation. Anesthesia was maintained with 2% sevoflurane and 0.1 µg/kg/min remifentanil. Intraoperative ventilation remained stable with acceptable tidal volumes and peak inspiratory pressures. 

During the final phase of the operation, the surgical team attempted to place a nasogastric tube (NGT) to decompress the stomach. Despite multiple attempts, the NGT repeatedly entered the trachea. Blood was noted on the first NGT, which was removed, and a second tube was advanced to 55-60 cm. Following these attempts, anesthesia noted a progressive decline in oxygen saturation to 89%, despite 100% fraction of inspired oxygen (FiO₂). On reassessment, ventilation was inadequate, with rising peak airway pressures (approximately 25 cm water (H₂O)), and auscultation revealed diminished breath sounds on the right. EtCO_2_ levels abruptly rose from the low 30s (30-34) to the high 40s, with a high of 61. 

An anesthesia stat team was activated. Direct laryngoscopy revealed copious blood in the oropharynx. This was then subsequently suctioned by the anesthesia team. An endotracheal tube was confirmed in the proper position by fiberoptic bronchoscopy. Despite confirmation of tube placement above the carina and the presence of end-tidal CO₂, oxygen saturation continued to decline to 82%. Breath sounds remained absent on the right. This indicated the placement of a right-sided chest tube. Following decompression, oxygen saturation improved to 100%. 

**Figure 1:**
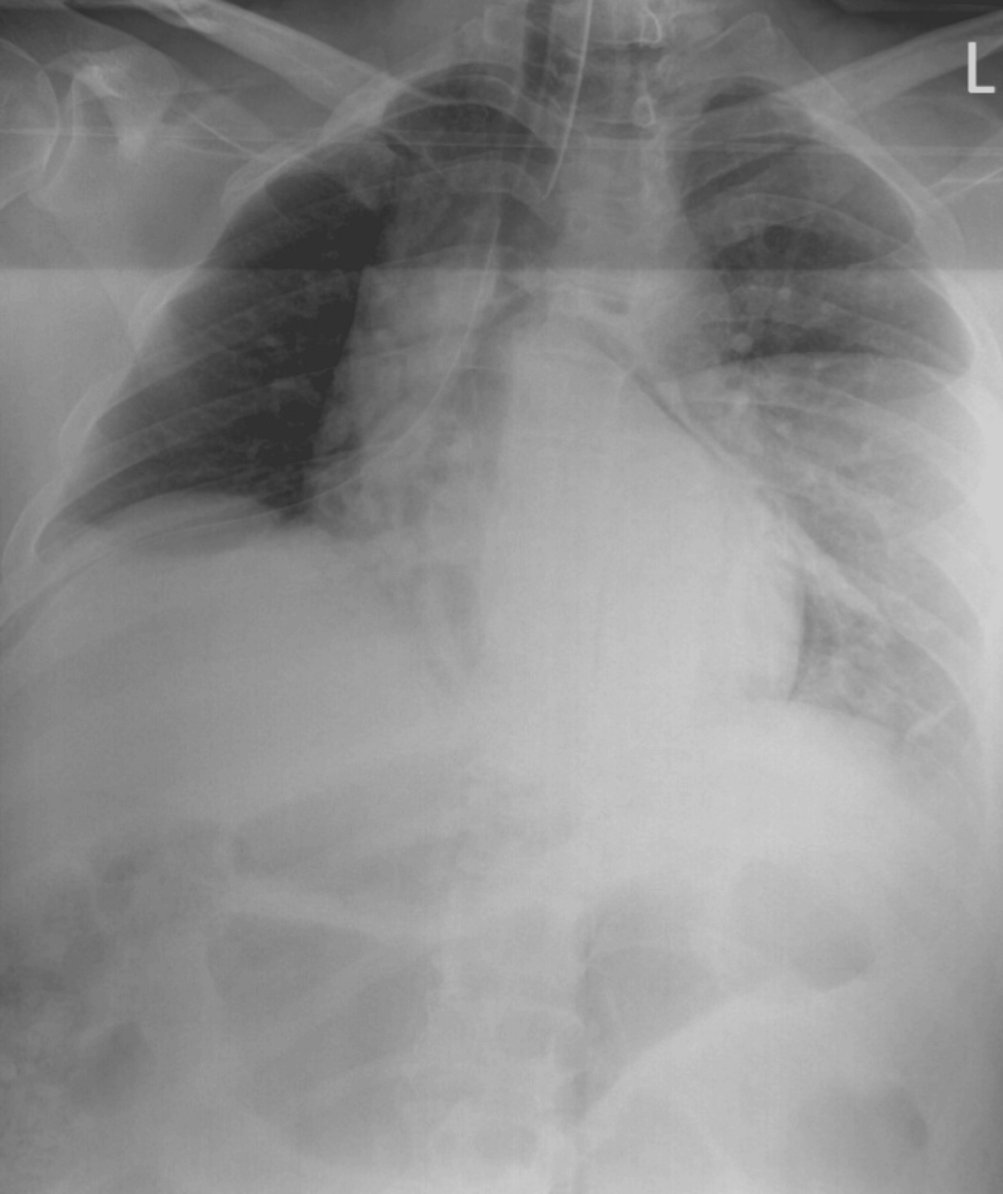
Chest X-ray obtained on postoperative day 0 X-ray completed in OR status post chest tube placement. The chest X-ray shows the tip of the right chest tube projects over the right upper mediastinum. The lungs are well expanded with asymmetric lung densities likely due to overlying soft tissue on the left hemithorax, due to patient rotation. There are bibasilar atelectatic changes noted. No definite pneumothorax is visualized.

Given the concern for tracheal injury from repeated NGT misplacement, the surgical team performed a bronchoscopy. The endotracheal tube was withdrawn under direct visualization. No tracheal disruption or injury was identified. The patient was subsequently extubated without complication. A chest X-ray was obtained in the OR status post chest tube, and no definite pneumothorax was visible (Figure 1).

**Figure 2:**
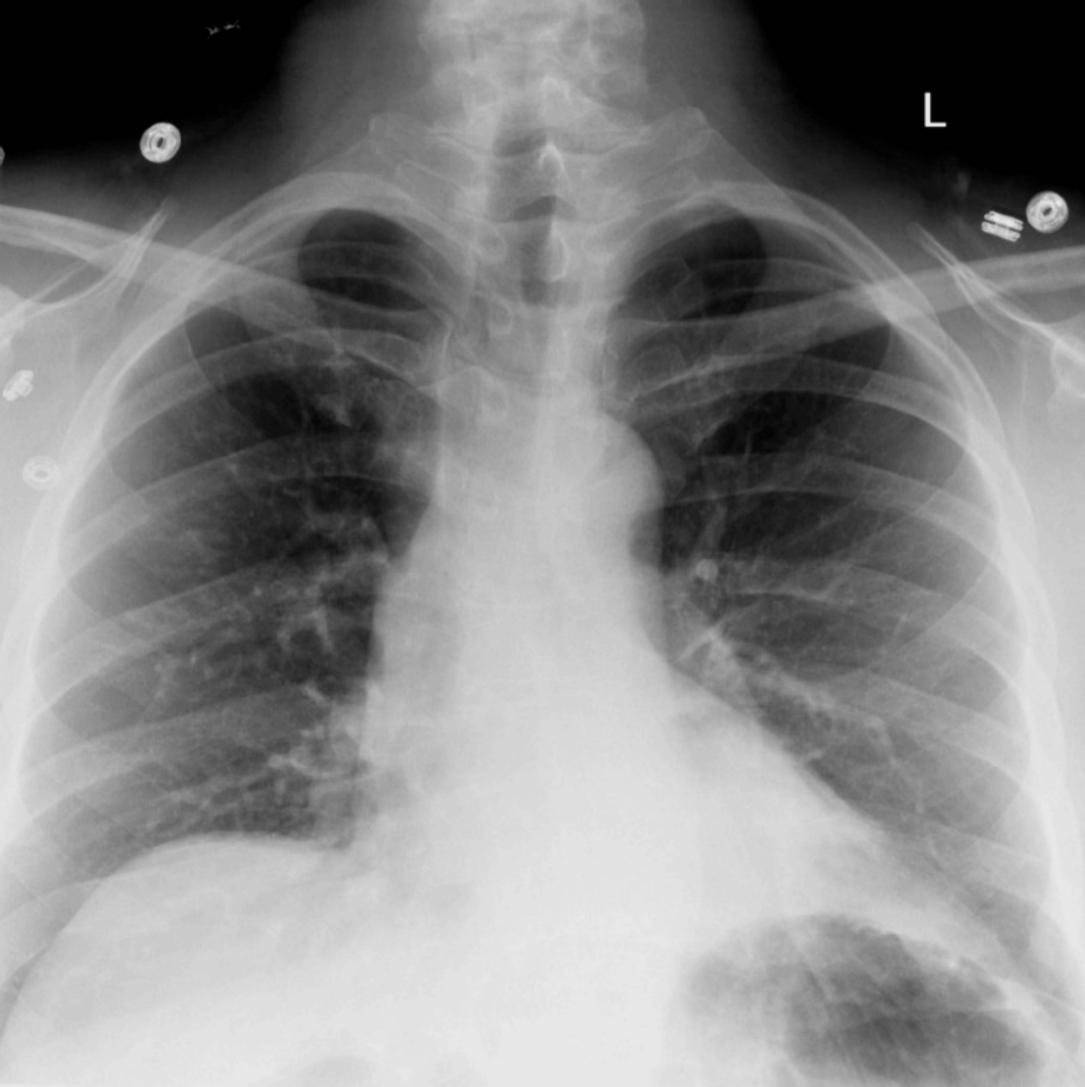
Chest X-ray obtained on postoperative day 1 after removal of the chest tube There is no evidence of a large pleural effusion or pneumothorax.

The patient was transferred to the post-anesthesia care unit (PACU) hemodynamically stable, with a pain score of 0 and oxygen saturation maintained on 15 L/min via a non-rebreather mask. A chest radiograph obtained that evening revealed a new 2 cm left-sided pneumothorax, in addition to the previously decompressed right-sided pneumothorax. The patient reported dyspnea and chest pain exacerbated by inspiration but remained otherwise stable. On postoperative day one, imaging demonstrated no evidence of a large pleural effusion or pneumothorax (Figure 2). The patient remained asymptomatic, with pain well controlled and oxygen saturation at 100%. The patient was discharged on postoperative day 2. 

## Discussion

We experienced the complex scenario of intrathoracic insufflation during an elective robotic-assisted laparoscopic hiatal hernia repair with Toupet fundoplication, which is a 270-degree partial wrap of the gastric fundus around the lower esophagus [1]. Toupet fundoplication has slightly lower rates of postoperative dysphagia and gas bloat and is a common alternative to Nissen fundoplication [1]. Despite the minor difference, the operative technique and anatomical exposure are nearly identical [1]. Since intrathoracic insufflation is more commonly described in the context of Nissen fundoplication, we refer to the broader literature accordingly.

Intrathoracic insufflation is a noteworthy complication that can arise during laparoscopic Nissen fundoplication for the treatment of GERD [6]. This unintended occurrence involves the introduction of insufflation gas, typically CO₂, into the thoracic cavity, leading to significant clinical challenges. The case report provides a detailed examination of the etiology, detection, management, and implications of intrathoracic insufflation, emphasizing the importance of awareness and preventive strategies during laparoscopic procedures. 

Laparoscopic Nissen fundoplication is esteemed for its effectiveness in treating GERD by reinforcing the lower esophageal sphincter through the wrapping of the gastric fundus around the lower esophagus [2]. However, the precision required in this minimally invasive surgery means that any deviation can result in complications such as intrathoracic insufflation [5]. The inadvertent entry of CO₂ into the thoracic cavity can be attributed to anatomical variations, such as congenital diaphragmatic defects, or technical errors during trocar placement or tissue manipulation. Intrathoracic insufflation can manifest as pneumothorax, pneumomediastinum, or subcutaneous emphysema [6]. The incidence rates for these complications vary, with pneumothorax occurring in approximately 1.9% of cases, subcutaneous emphysema in 2.3%, and pneumomediastinum in 1.9% [6]. Another study with 538 antireflux operations and 92 complications shows pneumothorax to have a 2.4% incidence rate [7]. Increased intra-abdominal pressure from CO₂ insufflation can lead to increased intrathoracic pressure, which may contribute to these complications [8]. It is essential to monitor patients closely for signs of these complications, especially in those with predisposing factors. 

The etiology of intrathoracic insufflation during laparoscopic procedures is multifactorial. It may stem from anatomical variations, such as congenital diaphragmatic defects [9], or from technical errors during trocar placement or tissue manipulation. This can inadvertently lead to diaphragmatic or pleural injury, resulting in migration of insufflated gas into the pleural cavity and causing pneumothorax [10]. The recognition and management of intrathoracic insufflation are critical for ensuring patient safety and optimizing surgical outcomes.  

Early detection of intrathoracic insufflation is paramount for ensuring patient safety and optimizing surgical outcomes. Intraoperative monitoring methods, such as analysis of end-tidal CO₂ levels, respiratory mechanics, and breath sound identification of the thoracic cavity,y are essential for early recognition. The case report emphasizes that vigilant monitoring can facilitate timely intervention, thereby mitigating the risks associated with intrathoracic insufflation. The crucial three signs seen in our case are an abrupt increase in end-tidal CO₂, sudden desaturation, and absent breath sounds. The first, an abrupt increase in end-tidal CO2, could be due to CO_2_’s highly diffusible nature and its tendency to get rapidly absorbed by the blood from the pleural space [11], which leads to a rapid rise in arterial and exhaled CO_2_. Furthermore, a decrease in minute ventilation can further propagate the issue. Another clinical sign that can be seen in this scenario, highlighted in our case, is the increase in peak airway pressures, which refers to an increased pressure needed to ventilate the lung. The ventilator requires higher pressure to achieve the same tidal volume and is a measure of airway resistance [12]. This is a highly valuable clinical indicator to diagnose a pneumothorax, especially a tension pneumothorax [13]. Finally, the absence of breath sounds is a critical clinical sign that indicates that the lung is not properly being ventilated. In an intrathoracic insufflation progressing into tension pneumothorax etiology, it is most likely due to the CO₂ in the air space compressing the lung, preventing ventilation [13]. 

Management strategies for intrathoracic insufflation include immediate cessation of insufflation, appropriate decompression techniques, and potentially transitioning to open surgery if necessary. Management in our case aligned closely with established protocols for intraoperative pneumothorax. Upon recognizing signs of ventilatory compromise and progressive hypoxia, the anesthesia team initiated a stat response and promptly confirmed endotracheal tube positioning using fiberoptic bronchoscopy. For decompression, a right-sided chest tube [14][15] was placed without delay or reliance on imaging, leading to immediate improvement in oxygen saturation. Simultaneously, insufflation was halted, and the pneumoperitoneum was deflated to prevent further gas migration into the pleural space. This decision to intervene early likely prevented further clinical deterioration, and there was no need for open surgery. Of note, a very crucial element in this case is the resolution of symptoms due to prompt treatment. Due to CO₂’s high solubility compared to room air, once insufflation is stopped and the gas is vented or absorbed, the lung can potentially re-expand and prevent decompensation into a critical tension etiology with severe compression of mediastinal structures [11]. This highlights a potential benefit in the immediate cessation of insufflation with rapid intervention in preventing morbidity. 

This case underscores the value of decisive action based on clinical findings and highlights the importance of seamless collaboration between anesthesia and surgical teams. Additionally, preventive strategies such as meticulous trocar placement, careful tissue manipulation, and thorough preoperative assessment of anatomical variations can significantly reduce the risk of intrathoracic insufflation.

## Conclusions

The case report on intrathoracic insufflation during laparoscopic Toupet fundoplication provides a comprehensive analysis of this significant complication. Through detailed examination of its causes, detection, and management, the report highlights the importance of vigilance, prompt intervention, and preventive measures. Surgeons can benefit from these insights, enhancing their technique and ultimately improving patient safety and surgical success. 

## References

[1] Frazzoni M, Piccoli M, Conigliaro R, Frazzoni L, Melotti G (2014). Laparoscopic fundoplication for gastroesophageal reflux disease. World J Gastroenterol.

[2] Seeras K, Bittar K, Siccardi MA (2025). Nissen Fundoplication. https://www.ncbi.nlm.nih.gov/books/NBK519521/.

[3] Dallemagne B, Weerts JM, Jehaes C, Markiewicz S, Lombard R (1991). Laparoscopic Nissen fundoplication: preliminary report. Surg Laparosc Endosc.

[4] Singhal T, Balakrishnan S, Hussain A, Grandy-Smith S, Paix A, El-Hasani S (2009). Management of complications after laparoscopic Nissen's fundoplication: a surgeon's perspective. Ann Surg Innov Res.

[5] Yadlapati R, Hungness ES, Pandolfino JE (2018). Complications of antireflux surgery. Am J Gastroenterol.

[6] Murdock CM, Wolff AJ, Van Geem T (2000). Risk factors for hypercarbia, subcutaneous emphysema, pneumothorax, and pneumomediastinum during laparoscopy. Obstet Gynecol.

[7] Pohl D, Eubanks TR, Omelanczuk PE, Pellegrini CA (2001). Management and outcome of complications after laparoscopic antireflux operations. Arch Surg.

[8] Kamine TH, Elmadhun NY, Kasper EM, Papavassiliou E, Schneider BE (2016). Abdominal insufflation for laparoscopy increases intracranial and intrathoracic pressure in human subjects. Surg Endosc.

[9] Azocar RJ, Rios JR, Hassan M (2002). Spontaneous pneumothorax during laparoscopic adrenalectomy secondary to a congenital diaphragmatic defect. J Clin Anesth.

[10] Machairiotis N, Kougioumtzi I, Dryllis G (2014). Laparoscopy induced pneumothorax. J Thorac Dis.

[11] Phillips S, Falk GL (2011). Surgical tension pneumothorax during laparoscopic repair of massive hiatus hernia: a different situation requiring different management. Anaesth Intensive Care.

[12] Mora Carpio AL, Mora JI (2025). Ventilator Management. https://www.ncbi.nlm.nih.gov/books/NBK448186/.

[13] Thachuthara-George J (2021). Pneumothorax in patients with respiratory failure in ICU. J Thorac Dis.

[14] Ravi C, McKnight CL (2025). Chest Tube. https://www.ncbi.nlm.nih.gov/books/NBK459199/.

[15] Heyba M, Rashad A, Al-Fadhli AA (2020). Detection and management of intraoperative pneumothorax during laparoscopic cholecystectomy. Case Rep Anesthesiol.

